# Beyond Antibiotics: One Health Education for Tackling Antimicrobial Resistance

**DOI:** 10.3390/antibiotics15070677

**Published:** 2026-07-10

**Authors:** Beatriz Robredo

**Affiliations:** OneHealth-UR Research Group, Area of Didactics of Experimental Sciences, Department of Agriculture and Food, University of La Rioja, 26006 Logroño, Spain; beatriz.robredo@unirioja.es; Tel.: +34-941-299724

**Keywords:** educational programmes, resources, science education, health behaviour, antibiotic stewardship, public health, health literacy

## Abstract

Antimicrobial resistance (AMR) is recognized as one of the most urgent global health threats, demanding coordinated, multisectoral responses under the OH framework. Among the multidisciplinary tasks aimed at collectively tackling the AMR crisis, surveillance, research and education stand as major priorities. Education is a strategic pillar of the World Health Organization Global Action Plan, yet a comprehensive synthesis of educational initiatives explicitly grounded in One Health (OH) remains limited. Previous reviews have examined AMR educational interventions focusing on specific strategies, regions, professional groups, or pedagogical tools, but without an OH perspective. This review is the first to comprehensively synthesize educational programmes that explicitly integrate OH principles across different educational levels, target audiences, and learning settings. It also examines the pedagogical strategies used to promote AMR awareness, prevention, and responsible antimicrobial use. A structured literature search (2015–2025) was conducted in Scopus and complemented by institutional sources and citation tracking. Educational initiatives incorporating OH principles, addressing multiple sectors, or promoting interdisciplinary AMR education were narratively synthesized. School-based programmes (e.g., e-Bug, ISGlobal initiatives, Ambientech); public awareness and community education via national strategies such as PRAN; programmes for university students; professional training, and continuing education (e.g., ESCMID, AMR EDUCare); and international online platforms including FAO e-learning programmes and the OH Workforce Academy were examined. Programmes were analysed according to target population, pedagogical approach, sectoral integration, and evaluation methods. Active and experiential methodologies, such as service-learning (e.g., Tiny Earth, MicroMundo) game-based learning, gamification, and interdisciplinary and systems thinking-based learning, consistently enhance knowledge acquisition, systems thinking skills, and awareness of cross-sectoral AMR transmission pathways. Despite all these initiatives, studies on knowledge, perceptions and attitudes about AMR point to clear errors and deficiencies. Key gaps, such as inconsistent curriculum integration, limited integration of environmental dimension and scarce rigorous impact evaluations, persist. Strengthening OH-oriented AMR education requires policy-level curriculum inclusion, cross-sector collaboration, standardized competencies, and robust evaluation frameworks. Embedding education within national AMR strategies is essential to fostering sustained behavioural change and preserving antimicrobial effectiveness across human, animal, and environmental systems.

## 1. Introduction

### 1.1. The Global AMR Crisis

Antimicrobial resistance (AMR) has emerged as one of the most significant threats to global public health in the 21st century. The World Health Organization (WHO) identifies AMR as one of the top ten global public health threats facing humanity, with projections estimating that antibiotic-resistant infections could cause 10 million deaths annually by 2050 if no effective interventions are implemented [1,2]. In Spain alone, antibiotic resistance is responsible for approximately 4000 deaths per year, while across Europe, this figure reaches 33,000 annual fatalities [3].

Beyond its impact on morbidity and mortality, AMR has profound social and economic consequences worldwide. Low- and middle-income countries are disproportionately affected due to limited healthcare infrastructure, inadequate sanitation, weak surveillance systems, unrestricted access to antimicrobials, and persistent educational inequalities, all of which facilitate the emergence and spread of resistant microorganisms. AMR also threatens food security and livestock production by reducing the effectiveness of antimicrobial treatments in animal health and increasing production losses, while imposing substantial costs on healthcare systems through prolonged hospitalizations, more complex treatments, and reduced productivity. These challenges reinforce the need for comprehensive educational strategies that promote antimicrobial stewardship and OH competencies across all sectors [4].

The emergence and spread of antibiotic resistance are driven by multiple interconnected factors, including inappropriate antibiotic use in human medicine, overuse in veterinary medicine and agriculture, and environmental contamination through pharmaceutical waste and hospital effluents [5,6]. In addition, antimicrobial use in crop production, food production systems, and aquaculture contributes to the selection and dissemination of resistant microorganisms, while international food trade facilitates their global spread. Resistant microorganisms and resistance genes may circulate through interconnected human, animal, plant, food, and environmental pathways, highlighting the complexity of AMR transmission. This complex, multifaceted nature of AMR necessitates a comprehensive, integrated approach that transcends traditional sectoral boundaries [7].

### 1.2. The One Health (OH) Framework

The OH approach provides a critical framework for addressing AMR by recognizing the intrinsic interconnections between human health, animal health, plant health, food safety, and environmental health [8,9]. According to the One Health High-Level Expert Panel (OHHLEP), One Health is an integrated, unifying approach that aims to sustainably balance and optimize the health of people, animals, plants, and ecosystems by recognizing that the health of humans, domestic and wild animals, plants, and the wider environment are closely linked and interdependent. This broader perspective is reflected in the current One Health framework promoted by the Quadripartite collaboration (WHO, FAO, WOAH, and UNEP), which expands the traditional human–animal–environment paradigm to encompass five interconnected dimensions: human health, animal health, plant health, food safety, and environmental health [10].

This holistic perspective is essential for developing effective strategies to combat AMR, as resistant bacteria and resistance genes can be transmitted among humans, animals, plants, food products, and environmental reservoirs through multiple interconnected pathways, including zoonotic transmission, food chains, contaminated water systems, wildlife, and direct contact [11,12]. These interconnected sectors facilitate the emergence and dissemination of antimicrobial resistance through food production systems, environmental contamination, and the movement of resistant microorganisms and resistance genes across humans, animals, plants, food products, and ecosystems, with zoonotic transmission representing one of the major pathways linking these sectors.

The scientific evidence supporting the OH approach to AMR is substantial.

Molecular epidemiological studies have demonstrated the transmission of antimicrobial-resistant bacteria and resistance genes among humans, animals, food products, and environmental reservoirs within the OH framework. Several examples of these transmission dynamics are highlighted in this Special Issue, “A One Health Approach to Antimicrobial Resistance”. Resistant *Staphylococcus* spp. and *Mammaliicoccus sciuri* strains carrying antibiotic resistance genes were identified in both poultry and healthy humans, suggesting potential interspecies dissemination through direct contact and food production systems [13]. Similarly, resistant Escherichia coli strains isolated from free-living birds were found to harbour virulence and resistance traits associated with human infections, emphasizing the role of wild birds as environmental reservoirs and vectors of antimicrobial resistance [14]. In addition, the detection of antibiotic-resistant bacteria in street foods and wastewater environments further supports the role of contaminated food products and environmental compartments in the dissemination of antimicrobial resistance to human populations [15,16].

Complementing these findings, recent work on wildlife sentinels has shown that red foxes (*Vulpes vulpes*) in Western Romania can harbour antimicrobial-resistant bacterial strains, reinforcing their role as effective indicators of AMR circulation at the wildlife–human interface [17]. Environmental studies from aquatic ecosystems in agricultural regions, including areas with intensive pig farming in Spain, have further demonstrated the presence and genetic diversity of multidrug-resistant Enterobacteriaceae in surface waters and wastewater treatment plants, highlighting the contribution of livestock-associated contamination to environmental AMR reservoirs [18]. Broader OH syntheses also emphasize the plasmid-mediated global spread of high-risk resistance determinants such as mcr genes conferring colistin resistance, illustrating how clinical, agricultural, and environmental compartments are interconnected through mobile genetic elements [19]. Similarly, large-scale reviews of dairy farm environments across Asia report consistent AMR patterns linked to antimicrobial use practices in livestock systems, reinforcing the agricultural contribution to environmental dissemination [20]. Collectively, these studies strengthen the concept that antimicrobial resistance is maintained and propagated through tightly linked human, animal, and environmental pathways, requiring integrated surveillance and mitigation strategies across all sectors.

Beyond zoonotic transmission, environmental contamination through wastewater, agricultural runoff, and pharmaceutical residues creates important reservoirs of antimicrobial resistance, while food production and distribution systems facilitate the movement of resistant microorganisms across geographical regions. Consequently, effective AMR mitigation requires integrated surveillance and coordinated interventions encompassing human, animal, plant, food, and environmental sectors under the One Health framework.

### 1.3. The Role of Education

Education plays a central role in implementing the OH approach to AMR. The WHO Global Action Plan on Antimicrobial Resistance identifies improved awareness through effective communication, education, and training as a fundamental pillar for combating resistance. Educating diverse stakeholders including students, healthcare professionals, veterinarians, farmers, policymakers, and the general public is essential for promoting responsible antibiotic use and implementing effective stewardship practices [1].

Despite the growing number of educational initiatives addressing AMR, a comprehensive synthesis of programmes explicitly based on the OH approach is still lacking. Existing reviews have not examined AMR education from an OH perspective, instead focusing on specific educational interventions, such as gamification [21] or online infographics [22], particular geographic regions, such as Africa [23], professional groups, such as veterinarians [24], or individual pedagogical tools [25].

This review aims to compile and analyse educational interventions on AMR developed within the OH framework, with a focus on formal and informal educational programmes targeting students, professionals, and communities. It also examines the pedagogical strategies used to promote AMR awareness, prevention, and responsible antimicrobial use, while providing an integrated overview of current levels of AMR knowledge and awareness among students and the general public.

Ultimately, this work seeks to inform and support researchers, educators, and policymakers in the design, implementation, and improvement of effective AMR education strategies.

## 2. Methodological Approach of the Review

This manuscript presents a comprehensive narrative review based on a structured literature search. Searches were conducted in Scopus in December 2025, covering the period from 2015 to 2025, corresponding to the adoption of the World Health Organization Global Action Plan on Antimicrobial Resistance and the subsequent expansion of OH-oriented AMR initiatives. The search strategy combined terms related to “OH”, “antimicrobial resistance”, and “education”, yielding 210 references.

In addition to the references identified through the database search, supplementary sources were identified through citation tracking and targeted searches of relevant institutional websites, including those of international organisations involved in AMR and OH education (e.g., WHO, FAO, WOAH, ECDC, and national AMR strategies). This approach enabled the inclusion of educational programmes, training resources, and policy documents that are often not represented in bibliographic databases.

Studies and programmes were included if they met at least one of the following criteria: (i) explicitly referenced the OH approach; (ii) addressed AMR across more than one sector (human, animal, or environmental health); or (iii) promoted interdisciplinary education, training, or collaboration related to antimicrobial use and resistance.

Educational initiatives targeting diverse audiences were considered, including healthcare and veterinary professionals, farmers, policymakers, students, and the general public. Both formal educational settings (school programmes, university curricula, and professional training) and informal approaches (community campaigns, online courses, and public awareness initiatives) were included. Data were synthesized narratively to provide a comprehensive overview of the current landscape of OH-oriented AMR education.

Overall, the review drew on approximately 250 documentary sources, including peer-reviewed publications, institutional reports, policy documents, and educational resources. The narrative synthesis examined 17 major educational programmes and initiatives, together with more than 20 additional educational resources, online courses, repositories, and public awareness activities covering school, university, professional, and community education.

Given the heterogeneous nature of the included sources (peer-reviewed articles, reports, educational resources, and institutional websites), no formal quality assessment tool was applied. This limitation has been acknowledged.

## 3. Political and Technical Context of the OH Approach

### 3.1. Key Political and Technical Milestones (2015–2025)

**Figure 1:**
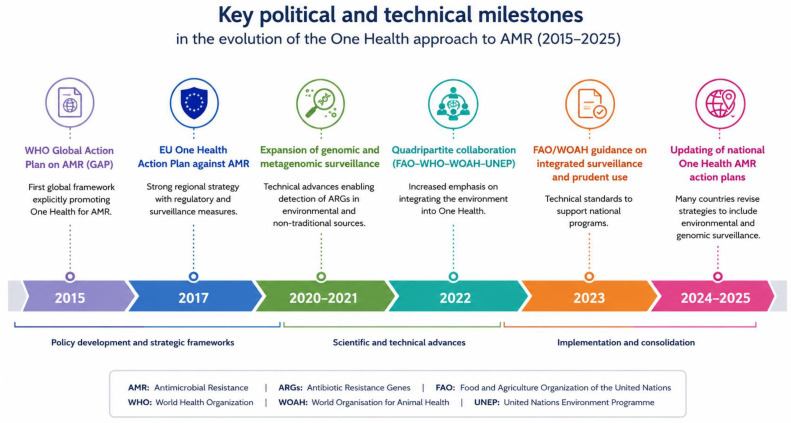
Timeline of the major political and technical milestones that have shaped the evolution of the OH approach to AMR (2015–2025).

The evolution of the OH approach in the context of AMR has been marked by important political and technical milestones that have shaped global and national responses to antimicrobial resistance (Figure 1).

2015: WHO Global Action Plan on AMR

The World Health Organization (WHO) Global Action Plan on Antimicrobial Resistance (AMR) represented the first global policy framework to explicitly adopt a One Health approach for tackling AMR. This landmark document established five strategic objectives that integrate human, animal, and environmental sectors: (i) improving awareness and understanding of AMR; (ii) strengthening knowledge through surveillance and research; (iii) reducing infection incidence; (iv) optimizing antimicrobial use; and (v) ensuring sustainable investment in AMR responses. The plan emphasized the critical importance of education and awareness-raising across all sectors.

From an educational perspective, the Global Action Plan established awareness and education as core components of AMR mitigation. It provided the policy foundation for the development of educational initiatives targeting healthcare professionals, veterinarians, students, policymakers, and the general public.

2017: A European One Health Action Plan Against AMR

The European Union adopted a comprehensive OH Action Plan against AMR, representing a strong regional strategy with regulatory and surveillance measures. This plan served as a model for other regions, demonstrating how coordinated policy frameworks can address AMR across human health, animal health, food production, and environmental sectors simultaneously. The EU plan included specific provisions for the education and training of healthcare professionals, veterinarians, and the public.

The plan strengthened the role of education within AMR policies by promoting capacity building and professional training, while encouraging Member States to integrate AMR awareness into public health and educational strategies.

2020–2021: Expansion of genomic and metagenomic surveillance

The World Organisation for Animal Health (OIE; renamed WOAH in 2022) released updated recommendations on animal AMR surveillance and veterinary practices, providing technical standards to support national programmes. Simultaneously, significant technical advances enabled detection of antibiotic resistance genes (ARGs) in environmental and non-traditional sources through expansion of genomic and metagenomic surveillance capabilities. These technological developments allowed for more comprehensive monitoring of resistance dissemination across the OH continuum and provided evidence for educational programmes about environmental dimensions of AMR.

These advances expanded the knowledge base available for education and training, supporting the inclusion of genomic surveillance, environmental reservoirs of resistance, and data interpretation skills in OH educational programmes.

2022: Expansion of Quadripartite Collaboration

In 2022, the long-standing Tripartite collaboration between the Food and Agriculture Organization (FAO), WHO, and WOAH (formerly OIE) was expanded through the inclusion of UNEP, forming the Quadripartite. This expansion recognized that environmental contamination and ecological factors play crucial roles in the emergence, persistence, and spread of antimicrobial resistance. The incorporation of UNEP and the widespread recognition of the broader One Health definition proposed by the One Health High-Level Expert Panel (OHHLEP) reinforced the inclusion of plant health, food systems, and environmental dimensions within OH strategies for AMR [7].

For AMR education, this milestone reinforced the need for interdisciplinary learning and highlighted the importance of incorporating environmental dimensions into training programmes that had traditionally focused on human and animal health.

2023: FAO/WOAH guidance on integrated surveillance and prudent use

FAO and WOAH released technical guidance on integrated surveillance and the prudent use of antimicrobials, providing technical standards to support national AMR programmes. These documents strengthened the implementation of OH strategies by promoting harmonized surveillance systems, antimicrobial stewardship, and cross-sector collaboration.

From an educational perspective, these technical guidelines highlighted the need for workforce training in integrated surveillance, prudent antimicrobial use, data interpretation, and interdisciplinary collaboration, supporting competency based OH education.

2024–2025: Updating of national OH AMR action plans

During 2024–2025, many countries revised their national One Health AMR action plans to incorporate environmental surveillance, genomic monitoring, and strengthened cross-sector governance, reflecting the continued evolution of One Health implementation.

This period represents the transition from policy development toward practical implementation, with educational programmes increasingly aligned with national AMR strategies and focused on developing competencies in antimicrobial stewardship, surveillance, systems thinking, and interdisciplinary collaboration.

Despite these important international milestones, the implementation of OH approaches to AMR remains uneven across countries. Low- and middle-income countries continue to face substantial challenges, including limited financial resources, inadequate laboratory and surveillance capacity, shortages of trained professionals, and insufficient integration of OH principles into education and workforce development [26,27]. Strengthening educational capacity, interdisciplinary training, and institutional collaboration in these settings will be essential to ensure that global OH policies can be effectively translated into national AMR strategies and sustainable educational programmes.

### 3.2. Educational Implications of OH Policies

Implementing AMR requires fundamental changes in education and training across multiple disciplines, as it is widely recognized in the scientific literature [7]. Healthcare professionals need to understand not only the clinical mechanisms of resistance, but also the environmental and agricultural drivers that contribute to its emergence and spread. In the same way, veterinarians must be aware of the implications of antimicrobial use in animals for human health, while farmers and agricultural workers require specific training on responsible antibiotic use and its environmental consequences. Environmental scientists also play a key role, as they must understand how antibiotic residues and resistance genes circulate within ecosystems and ultimately affect human and animal health systems [6].

From an educational perspective, this implies the need for truly interdisciplinary curricula that integrate human, animal and environmental health under a systems thinking framework [28]. For the general public and students, OH education is essential to foster health literacy and promote behavioral change. This includes appropriate antibiotic use, adherence to vaccination programmes, infection prevention practices, and environmental stewardship. Such competencies are consistently highlighted as crucial in global OH frameworks, which emphasize the importance of cross-sectoral education to effectively combat AMR [29]. Overall, the literature supports the idea that addressing AMR requires not only scientific and policy coordination, but also a profound transformation in how health-related knowledge is taught and understood across society.

### 3.3. Educational Landscape of AMR

Traditionally, AMR was not taught in relation to infectious diseases but rather addressed to understand evolutionary mechanisms. AMR has been used as a context to develop and assess students’ knowledge of natural selection, mutations, and evolution [30]. For instance, it has been used in virtual simulations to explore concepts related to natural selection and the generation of AMR [31]. Animations have also been used to help students overcome the notion that mutations originate in response to the environment and to antibiotics [32].

Currently, collaborative educational initiatives have been implemented, such as service-learning projects and open schooling experiences [33,34,35], which connect secondary and university students with experts in AMR. These approaches aim to strengthen students’ understanding of the topic through real-world, interdisciplinary contexts.

Despite these efforts, many studies still address AMR in a limited way. Even when health education is included, it tends to focus mainly on antibiotic use, without fully incorporating broader factors such as environmental and animal health or the OH perspective. A systematic review of 144 studies in medicine, pharmacy, nursing, dentistry, and veterinary undergraduate students concluded that teaching about antimicrobials is often superficial and called for a deeper integration of ecological and OH dimensions [36]. A review published in 2021 analysed initiatives aimed at addressing AMR among different audiences from an OH perspective. Nevertheless, the primary emphasis was not on the OH approach itself, but rather on the misuse of antibiotics in humans [35].

Some authors have begun to introduce more holistic perspectives by incorporating the OH approach into AMR education. For example, open schooling initiatives have included specific OH modules, which students found particularly engaging because they addressed the impact of AMR on animals, plants, and the environment—dimensions that were previously unfamiliar to them [37]. Nevertheless, these studies did not systematically assess how much students actually learned about the OH concept itself. Despite this limitation, such initiatives reflect a growing tendency to integrate the OH perspective into educational strategies. In the following section, “Educational Programmes and Resources,” these emerging approaches will be explored in greater detail.

In the same line, the minimum core curriculum established by the new Spanish educational legislation includes topics related to health, infectious diseases, antibiotics, and ecosystems. However, AMR is not explicitly identified as a specific curricular concept, and the OH approach does not appear as an official curricular framework at the national level [38]. Nevertheless, each autonomous community further develops and specifies these contents. For instance, in the Autonomous Community of La Rioja, a specific section has been incorporated in the third year of compulsory secondary education that directly addresses antibiotics, entitled ‘Reflection on Self-Medication and the Appropriate Use of Antibiotics,’ as well as indirect references through applied learning in upper secondary education (Baccalaureate). This represents clear legislative progress in this area, as Robredo & Torres (2021) reported that antibiotics were not mentioned at any point in the secondary education curriculum [39]. The new local legislation also incorporates the ‘OH’ approach, linking human, animal, and environmental health, as well as zoonoses and the emergence of new diseases.

With the exception of recent evidence from the United States showing that AMR and OH remain largely absent from mainstream secondary biology curricula [40], the literature provides little information on the integration of these topics into national curricula elsewhere. Most published studies describe isolated educational initiatives rather than curriculum-level implementation, which may indicate that these topics have yet to be systematically embedded within formal secondary education programmes.

Barriers to integrating the OH approach into antimicrobial resistance education are based on several issues:

First, educational programmes are often organized in isolation by discipline, limiting opportunities for interdisciplinary learning and collaboration. As a result, students may acquire knowledge about antimicrobial resistance within their own field but lack an understanding of the interconnected pathways that link human, animal, and environmental health.

Second, the environmental dimension of the OH approach remains underrepresented. While human and veterinary aspects are increasingly incorporated into curricula, environmental reservoirs of antimicrobial resistance, wastewater management, pollution from pharmaceutical production, and ecological transmission pathways receive far less attention.

Finally, AMR itself is a complex problem that requires systems thinking skills. Traditional educational approaches often struggle to convey the multidimensional and interconnected nature of resistance emergence and transmission.

Overcoming these barriers will require greater institutional commitment, curriculum reform, intersectoral collaboration, competency-based educational frameworks, and more rigorous evaluation of educational outcomes.

## 4. Educational Programmes and Resources

**Figure 2:**
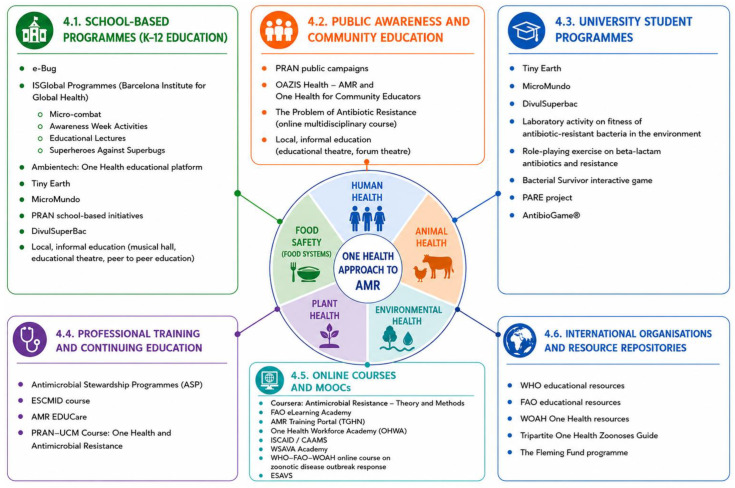
Classification of educational initiatives addressing AMR through an OH approach by target audience and educational setting.

This section presents the educational programmes and resources identified in this review. As shown in Figure 2, these initiatives are organized according to their target audience. 

To facilitate comparison, Table 1 summarizes their main characteristics, providing an overview of the educational approaches, pedagogical strategies, One Health integration, and available impact assessment data.

### 4.1. School-Based Programmes (K-12 Education)

The identified programmes and resources are described below. School-based programmes target students from primary through secondary education, providing age-appropriate content that builds foundational knowledge about microbes, infections, and antimicrobial resistance.

#### 4.1.1. e-Bug

Website: https://www.e-bug.eu/ (accessed on 29 May 2026).

e-Bug is an extensive international educational programme developed through European collaboration, with support from public health agencies including the European Centre for Disease Prevention and Control (ECDC). The programme provides comprehensive, evidence-based resources for teaching microbes, infection transmission, infection prevention, and responsible antibiotic use.

##### Programme Structure and Content

e-Bug offers age-appropriate materials for different educational stages, from primary education through secondary and higher education. For younger students (ages 5–11), resources focus on hand hygiene, how infections spread, and the basics of vaccination, using engaging activities like glow-germ demonstrations and interactive games. Secondary-level materials (ages 11–15) introduce more sophisticated concepts including antibiotic mechanisms, resistance development, and the distinction between bacterial and viral infections, a critical knowledge gap that contributes to inappropriate antibiotic use.

The program includes lesson plans, student worksheets, PowerPoint presentations, and practical laboratory activities. Notably, e-Bug incorporates hands-on microbiology experiments that allow students to culture bacteria, test antibiotic susceptibility, and observe resistance development firsthand. These experiential learning opportunities are particularly effective for conveying the reality of AMR.

##### OH Integration

Recent updates to e-Bug have strengthened its OH components. Materials now explicitly address antibiotic use in agriculture and its consequences for human health, environmental contamination from pharmaceutical waste, and zoonotic disease transmission. Case studies illustrate real-world OH scenarios, such as ESBL-producing *E. coli* transmission through contaminated food products or carbapenem-resistant bacteria in hospital wastewater affecting downstream environments. Evaluation studies have documented significant improvements in students’ knowledge and attitudes toward antibiotic use following e-Bug implementation, demonstrating the program’s effectiveness.

#### 4.1.2. ISGlobal Programmes (Barcelona Institute for Global Health)

Website: https://www.isglobal.org/en/antimicrobial-resistance (accessed on 29 May 2026).

The Barcelona Institute for Global Health (ISGlobal) has developed several notable educational initiatives addressing antibiotic resistance from a OH perspective.

##### Micro-Combat

Micro-Combat represents an innovative serious game approach to teaching antibiotic resistance. This card game, which received the 2018 award from the Spanish Agency of Medicines and Health Products for best awareness initiative on antibiotic resistance, engages players in the roles of healthcare professionals and researchers working to prevent bacterial infections. The educational value of Micro-Combat lies in its ability to make abstract concepts tangible through gameplay. Players directly experience how antibiotic overuse accelerates resistance development, how prevention strategies reduce the need for antibiotics, and how research investment is essential for developing new therapeutic options. The game is designed for ages 10 and above, making it suitable for secondary education contexts as well as public engagement activities.

##### Awareness Week Activities

During World Antimicrobial Awareness Week (WAAW), ISGlobal organizes educational installations and activities that illustrate bacterial growth and resistance development. These installations employ visual demonstrations, such as agar plate cultures showing resistant versus susceptible bacterial colonies, to make microbiological concepts accessible to general audiences. The activities explicitly connect human, animal, and environmental health under the OH framework, helping participants understand how resistance emerges and spreads across different settings.

##### Educational Lectures

ISGlobal researchers deliver educational talks in schools and community settings. These presentations cover the biological mechanisms of antibiotic resistance, the public health implications of AMR, and individual and collective actions to mitigate the problem. The lectures are tailored to different audiences, from primary school students to healthcare professionals, and emphasize the OH interconnections between different sectors.

##### ‘Superheroes Against Superbugs’

Website: https://educaixa.org/microsites/IS_GLobal/Superheroes_contra_superbacterias/ (accessed on 29 May 2026).

ISGlobal, in collaboration with “La Caixa Foundation” through the EduCaixa platform, developed Superheroes Against Superbugs, an interactive educational comic to raise awareness of antimicrobial resistance among secondary school students.

The comic is organized into several learning modules covering the discovery of penicillin, mechanisms of antibiotic action, the evolution and spread of bacterial resistance, and current research on new antimicrobial therapies.

#### 4.1.3. Ambientech: One Health Educational Platform

Website: https://ambientech.org/one-health (accessed on 29 May 2026).

Ambientech is a Spanish non-profit organisation comprising scientists and STEAM educators that develops interactive educational resources focused on science, health, and the environment for secondary education, vocational training, and the general public.

##### ‘OH: Una Sola Salud’ Resource

The flagship OH resource from Ambientech provides a comprehensive introduction to the interconnections between human, animal, and environmental health. Designed for secondary education and vocational training students, the resource employs animations and gamified activities to explain how diseases can transmit between animals and humans (zoonoses) and the factors that promote or prevent such transmission. The resource includes detailed case studies of two significant zoonoses: Lyme disease and echinococcosis (hydatidosis). For each disease, students learn about the pathogen’s life cycle, animal reservoirs, transmission routes to humans, clinical manifestations, and prevention strategies. These case studies effectively illustrate OH principles by demonstrating how wildlife management, domestic animal care, and human behaviour collectively influence disease risk.

##### Pedagogical Approach

Ambientech resources are distinguished by their active learning methodology. Interactive modules require students to make decisions, solve problems, and explore consequences, rather than passively receiving information. Gamification elements, such as points, badges, and progress tracking, enhance engagement and motivation. The platform’s comprehensive glossary serves as an additional learning support tool. All Ambientech resources are freely accessible online, removing financial barriers to adoption. The organisation also provides teacher guides and professional development opportunities to support effective classroom implementation.

#### 4.1.4. Tiny Earth: Crowdsourcing Antibiotic Discovery

Website: https://tinyearth.wisc.edu/ (accessed on 29 May 2026).

Tiny Earth is an innovative educational network engaging students in authentic antibiotic discovery research. Operating across 45 U.S. states and 15 countries, the program engages students in collecting soil samples from their local environments and screening bacterial isolates for antibiotic production capabilities. This ‘crowdsourcing’ approach serves dual purposes: advancing antibiotic discovery research while providing students with genuine research experiences. From an OH perspective, Tiny Earth is particularly valuable for illustrating environmental reservoirs of antibiotic-producing organisms and the ecological origins of antimicrobial compounds. Students gain hands-on experience with microbiological techniques, experimental design, and data analysis while contributing to addressing the global shortage of novel antibiotics. The programme has been successfully implemented in general biology, microbiology, and cell/molecular biology courses at various educational levels.

#### 4.1.5. MicroMundo

Website: https://www.ucm.es/small-world-initiative/proyecto (accessed on 29 May 2026).

MicroMundo is an educational and scientific project inspired by Tiny Earth that brings together universities and secondary/high school students to address AMR. The project is based on citizen science and service-learning. University students work with and mentor younger students to collect soil samples and isolate microorganisms that may produce new antibiotics. In this way, participants learn microbiology through hands-on activities while also contributing to real scientific research. Besides its scientific goals, MicroMundo promotes awareness of the OH concept, which connects human, animal, and environmental health, and encourages responsible antibiotic use. Over the past several years, many universities across Spain and Portugal have participated in the initiative, involving thousands of students in the collaborative search for new antibiotics and helping to increase interest in science and research [41].

#### 4.1.6. PRAN School-Based Initiatives

Website: https://resistenciaantibioticos.es/es (accessed on 29 May 2026).

Spain’s national action plan against antibiotic resistance (PRAN), coordinated by the Spanish Agency of Medicines and Health Products (AEMPS), collaborates with the Ministry of Education to integrate AMR education into school curricula. Teacher training workshops equip educators with knowledge and resources to teach antibiotic resistance effectively. Student materials, aligned with curriculum standards, cover topics including infection prevention, appropriate antibiotic use, the consequences of resistance, and OH interconnections.

#### 4.1.7. DivulSuperbac

The DivulSuperBac project was developed as an educational outreach initiative aimed at raising awareness on AMR and “superbugs” among pre-university students. The project engaged university students, particularly those in health-related degrees, through a service-learning approach that integrated academic learning with community service. The educational tool consisted of a traveling exhibition of 14 infographics called DivulSuperBac. The exhibition and related activities take place in secondary schools. Infographics are structured visual tools created to communicate complex scientific concepts in a clear, accessible, and engaging manner. They combine concise textual information with images, charts, and graphical elements to enhance understanding and retention of key messages related to AMR, disease control, and responsible antibiotic use; one out of the 14 infographics deals with the OH approach [42]. The infographics were originally developed at the University of Valencia, Spain, and the project is currently expanding to different universities and countries: Italy (Palermo), Poland (Opole), Germany (Mainz), Morocco (Fez), and Spain (Ourense, La Rioja) [43].

#### 4.1.8. Local Informal Educational Initiatives for K–12 Students

This includes activities for children such us the musical hall “The Mould that Changed the World” [40], the educational theatre “The drugs don’t work” [44], forum theatre on antibiotic use in Myanmar [45], school-based educational interventions on rational antibiotic use [46], debate activities [47] or peer-to-peer education about prudent antibiotic use in schools [48].

### 4.2. Public Awareness and Community Education

Community programmes translate complex scientific concepts into accessible messages for non-specialized audiences, targeting the general public through campaigns, events, and community-based initiatives.

#### 4.2.1. PRAN Public Campaigns

Website: https://resistenciaantibioticos.es/es (accessed on 29 May 2026).

PRAN has launched several public campaigns, including “Run Without Resistance”, consisting of running races and walks. Campaign materials include posters, videos, social media content, and informational brochures distributed through healthcare facilities, schools, and community centres. Educational materials explicitly adopt an OH framework, addressing antibiotic use in human medicine, veterinary medicine, and agriculture. Resources for healthcare professionals include clinical guidelines, continuing education modules, and stewardship toolkits. Materials for farmers and veterinarians focus on appropriate antimicrobial use in food-producing animals and the importance of infection prevention.

#### 4.2.2. OAZIS Health—AMR and OH for Community Educators

Website: https://oazis.rw/learn/courses/amr-and-one-health-for-community-educators/ (accessed on 29 May 2026).

OAZIS Health is an African-based e-learning platform, headquartered in Rwanda, focused on capacity building in public health and community health education. It offers the online course ‘AMR and OH for Community Educators’, which addresses antimicrobial resistance from a community oriented OH perspective. It introduces fundamental concepts of antimicrobial resistance, including causes, transmission pathways, and prevention strategies, emphasizing interconnections between human health, animal health, and the environment. The target audience includes community educators, health promoters, local leaders, and professionals involved in non-governmental or community-based health initiatives. Primarily oriented toward low- and middle-income country settings, it focuses on awareness-raising and behaviour change at the community level.

#### 4.2.3. Local Informal Education for Public Awareness and Community Engagement

This includes activities such as educational theatre and public discussions on antimicrobial resistance [49], forum theatre approaches for public engagement in Myanmar [45], and caregiver and children training sessions on rational antibiotic use [46].

#### 4.2.4. Online Multidisciplinary Course “The Problem of Antibiotic Resistance” for Teachers, Students and Citizens

The course includes videos and materials from different disciplines, such as microbiology, chemistry, and philosophy, helping participants understand the complexity of the problem from an OH perspective [50].

### 4.3. University Student Programmes

Some of the programmes mentioned in Section 4.1 and Section 4.2 are also suitable for university students. In other initiatives, such as Tiny Earth, MicroMundo, or DivulSuperbac, university students themselves act as disseminators of information, teaching and raising awareness among students at lower educational levels. The following are some specific activities designed for university students:

- Laboratory activity on fitness of antibiotic-resistant bacteria in the environment: This activity allows undergraduate students to investigate how antibiotic-resistant and sensitive bacteria survive under different environmental conditions. Students collect water samples from their communities and analyse bacterial fitness in the presence or absence of antibiotics. The activity promotes critical thinking, laboratory skills, and awareness of environmental antimicrobial resistance [51].
- Role-playing exercise on beta-lactam antibiotics and resistance: This kinaesthetic activity helps students understand how antibiotics work and how bacteria develop resistance. Students physically represent molecules and cellular structures, making abstract microbiological concepts easier to visualize and remember. The exercise encourages active participation and long-term retention of knowledge [52].
- Bacterial Survivor interactive game: This classroom game demonstrates how bacterial mutations and natural selection contribute to antibiotic resistance. Students participate in a fast-paced activity that challenges misconceptions about bacterial evolution and resistance mechanisms. It is especially effective in large biology or microbiology classes [53].
- The PARE Project (Prevalence of Antibiotic Resistance in the Environment) engages university students in environmental surveillance research. Students collect soil samples and test them for antibiotic-resistant bacteria using standardized microbiological methods. The project combines authentic scientific research with global collaboration and contributes to real antimicrobial resistance monitoring efforts [54].
- AntibioGame^®^ serious game for medical students: This educational game uses clinical scenarios to train medical students in responsible antibiotic prescription practices. Through role-playing and problem-solving activities, students improve their clinical decision-making skills and their understanding of antimicrobial stewardship in primary healthcare settings [55].

### 4.4. Professional Training and Continuing Education

Healthcare professionals play a central role in antimicrobial stewardship, infection prevention and control, and patient education. Strengthening their understanding of AMR during training is therefore essential to improve prescribing practices, adherence to infection control protocols, and public awareness [56,57]. Professional training programmes target healthcare workers, veterinarians, and other professionals with direct responsibilities in antimicrobial management, providing specialized knowledge and practical skills for their specific sectors.

In Spain, healthcare professionals such as physicians, pharmacists, nurses, and veterinarians are legally required to be registered with their corresponding professional regulatory bodies (colegios profesionales) in order to practice their profession. Membership involves the payment of professional fees and guarantees adherence to the ethical, legal, and professional standards established by these organisations. Beyond their regulatory role, professional colleges play an important function in continuing professional development by offering accredited training programmes, scientific updates, and educational activities in areas of major public health concern, including AMR and antimicrobial stewardship.

These professional organisations contribute significantly to the dissemination of knowledge and best practices regarding the prudent use of antibiotics. Continuing education initiatives promoted by professional colleges frequently include courses, workshops, seminars, and online training focused on rational antibiotic prescribing, infection prevention, and the reduction in antimicrobial resistance. This lifelong learning approach is particularly relevant because healthcare professionals are key actors in combating AMR through appropriate prescription practices, patient education, and interdisciplinary collaboration.

Furthermore, the involvement of professional regulatory bodies helps standardize competencies related to antimicrobial stewardship across healthcare disciplines. In Spain, this coordinated educational structure strengthens national and regional strategies aimed at addressing AMR, ensuring that healthcare professionals remain updated on evolving scientific evidence, clinical guidelines, and public health recommendations. Such institutional support reinforces the integration of AMR education not only during undergraduate training but also throughout professional practice, which is considered essential for an effective and sustainable OH response to AMR.

#### 4.4.1. Antimicrobial Stewardship Programmes (ASPs)

Antimicrobial Stewardship Programmes (ASPs) are coordinated institutional interventions designed to measure and improve the appropriate use of antimicrobials. Their primary goals are to enhance patient outcomes, reduce microbial resistance, decrease healthcare costs, and minimize adverse drug events.

To ensure a successful and sustainable framework, the Centers for Disease Control and Prevention (CDC) outline seven essential pillars for hospital ASPs: (i) Hospital Leadership Commitment—dedicating necessary human, financial, and technological resources; (ii) Accountability—appointing a single leader (often an infectious diseases physician) responsible for programme outcomes; (iii) Pharmacy Expertise—appointing a co-leader pharmacist specialized in infectious diseases to manage dosing and selection; (iv) Action—implementing specific interventions, such as systemic “antibiotic timeouts” or pre-authorization for restricted drugs; (v) Tracking—monitoring prescribing patterns, local resistance rates, and overall antimicrobial consumption; (vi) Reporting—regularly sharing use and resistance data with doctors, nurses, and hospital leadership; and (vii) Education—providing continuous training to healthcare workers regarding optimal prescribing practices.

While initially developed for high-acuity hospital settings, ASP principles apply across the entire healthcare spectrum: (i) Acute Care Hospitals—focus on severe infections, critical care dosing, and reducing healthcare-associated infections; (ii) Long-Term Care Facilities—tailored to elderly populations, heavily focusing on preventing the overtreatment of asymptomatic bacteriuria; and (iii) Outpatient and Primary Care—target public education and reduce unnecessary antibiotic prescriptions for viral upper respiratory tract infections.

ASPs guide clinicians to evaluate every prescription against five fundamental criteria: (i) Right Drug—selecting the most narrow-spectrum agent effective against the suspected or confirmed pathogen; (ii) Right Dose—tailoring the dosage to patient-specific factors like weight, age, organ function (especially kidney health), and site of infection; (iii) Right Duration—limiting the therapy to the shortest effective timeframe proven by clinical guidelines to avoid driving resistance; (iv) Right Route—preferring oral medications whenever clinically feasible, or executing an early intravenous-to-oral switch; and (v) De-escalation—reviewing microbiological culture results at 48–72 h to transition from broad-spectrum empiric therapy to targeted narrow-spectrum drugs.

#### 4.4.2. European Society of Clinical Microbiology and Infectious Diseases (ESCMID)

Website: https://www.escmid.org/education/education-ondemand/academy/an-introduction-to-antimicrobial-resistance/ (accessed on 29 May 2026).

The online course includes integrated modules on antimicrobial resistance, surveillance systems, policy frameworks, and selected OH considerations. The OH approach is limited but relevant, with emphasis on clinical and public health aspects. The target audience includes professionals in microbiology, medicine, and public health.

#### 4.4.3. AMR EDUCare (European Project)

Website: https://www.amreducare.eu/ (accessed on 29 May 2026).

These online educational materials and modular training courses are organized by the EU4Health Programme, coordinated by the Spanish Agency of Medicines and Medical Devices (AEMPS) with participation of 14 European partner organisations. Primarily it is centred on optimizing antimicrobial prescribing and clinical practices, with a limited but relevant OH dimension. The target audience includes healthcare professionals in antimicrobial resistance, focusing on prudent antibiotic use, educational strategies, and competencies aimed at improving clinical practice.

#### 4.4.4. PRAN–UCM Course: ‘OH and Antimicrobial Resistance’

Website: https://resistenciaantibioticos.es/es/lineas-de-accion/formacion/cursos/one-health-y-la-resistencia-los-antibioticos (accessed on 29 May 2026).

The course ‘OH and Antimicrobial Resistance’ is organized by the Spanish National Action Plan on Antimicrobial Resistance (PRAN) in collaboration with Universidad Complutense de Madrid (UCM), as part of UCM Summer Courses. This course represents a model of collaboration between health authorities and academic institutions for continuing professional education. Topics covered include the current AMR situation from an OH perspective, scientific and social challenges related to AMR, and control strategies and educational actions.

### 4.5. Online Courses and MOOCs (Open Access)

Massive Open Online Courses (MOOCs) and e-learning platforms have democratized access to AMR and OH education, reaching global audiences across diverse sectors and geographic regions with free or low-cost comprehensive training.

#### 4.5.1. Coursera: Antimicrobial Resistance—Theory and Methods

Website: https://www.coursera.org/learn/antimicrobial-resistance#modules (accessed on 29 May 2026).

This online course provides theoretical foundations and methodological tools for understanding AMR. The objective is to equip learners with basic analytical and conceptual tools to study AMR, including cross-sectoral surveillance approaches. It includes a dedicated module on OH and integrated surveillance systems linking human, animal, and environmental health. A certificate is available with the paid option.

#### 4.5.2. FAO eLearning Academy on Antimicrobial Resistance

Website: https://elearning.fao.org/course/view.php?id=783 (accessed on 29 May 2026).

The FAO offers several free online courses: “Understanding Antimicrobial Resistance in Food and Agriculture” (an introduction to AMR addressing human, animal, and environmental dimensions); ‘Raising Awareness on the Responsible Use of Antibiotics in Livestock’ (focused on animal production systems and prudent antimicrobial use); “FAO Progressive Management Pathway for AMR” (an OH-oriented management framework supporting policy development and implementation); and “FAO OH Course” (self-paced online course introducing the OH approach and its application to emerging infectious diseases, AMR, climate change, and food systems). These courses are designed for professionals, policymakers, and educators, with content available in multiple languages including Spanish and English.

#### 4.5.3. AMR Training Portal—The Global Health Network (TGHN)

Website: https://amr.tghn.org/training/ (accessed on 29 May 2026).

The AMR Training Portal of The Global Health Network offers a collection of free online courses addressing multiple aspects of antimicrobial resistance, including introduction to AMR, surveillance systems, responsible use of antibiotics, and OH and AMR. Courses are available in multiple languages, and selected modules explicitly integrate a multisectoral OH perspective.

#### 4.5.4. One Health Workforce Academy (OHWA): Antimicrobial Resistance in OH

Website: https://onehealthworkforceacademies.org/training/antimicrobial-resistance-2/ (accessed on 29 May 2026).

The OH Workforce Academy (OHWA) offers educational materials, applied learning activities, knowledge assessments and online training courses on antimicrobial resistance explicitly framed within an OH approach. The programme includes modules covering antimicrobial agents, mechanisms of resistance, rational use of antibiotics, public health impact, and surveillance across human, animal, and environmental sectors. There are introductory and advanced levels available.

#### 4.5.5. International Society for Companion Animal Infectious Diseases (ISCAID)

Website: https://www.iscaid.org/ (accessed on 29 May 2026).

The International Society for Companion Animal Infectious Diseases runs the CAAMS (Companion Animal Antimicrobial Stewards) committee. They host international webinars, workshops, and case-study sessions tailored specifically to resolving everyday clinical prescribing dilemmas in dog and cat veterinary practices.

#### 4.5.6. World Small Animal Veterinary Association Academy

Website: https://wsava.org/wsava-academy/ (accessed on 29 May 2026).

The World Small Animal Veterinary Association offers online modules and hybrid courses (often in collaboration with the World Organisation for Animal Health—WOAH) focused on why ASP matters in pets and how to apply global prescribing guidelines in private practice.

#### 4.5.7. WHO–FAO–WOAH Online Course on Zoonotic Disease Outbreak Response

Website: https://www.who.int/news/item/31-10-2023-fao--who--and-woah-launch-new-online-course-on-joint-response-to-zoonotic-disease-outbreaks (accessed on 29 May 2026).

This joint online training developed by the WHO, FAO, and WHOAH is focused on coordinated OH responses to zoonotic outbreaks.

#### 4.5.8. European School for Advanced Veterinary Studies (ESAVS)

Website: https://esavs.org/?utm_source=chatgpt.com (accessed on 29 May 2026).

The offered postgraduate veterinary education programmes integrate AMS and responsible antimicrobial use into specialties such as internal medicine, dermatology, and critical care.

### 4.6. International Organisations and Resource Repositories

International health organisations provide comprehensive educational resources, policy guidance, and technical materials that serve as valuable references for educators developing local or national educational programmes.

#### 4.6.1. World Health Organisation (WHO) Educational Resources

Website: https://openwho.org/ (accessed on 29 May 2026).

It provides comprehensive educational resources on AMR for diverse audiences. These include webinars, training modules, policy briefs, and communication materials available in multiple languages. Resources emphasize the OH approach and cover topics such as antimicrobial stewardship in healthcare and agriculture, surveillance systems, infection prevention and control, and regulatory frameworks. These international resources serve as valuable references for educators developing local or national educational programmes. They provide scientifically rigorous information reflecting current global consensus on AMR challenges and solutions.

#### 4.6.2. Food and Agriculture Organization (FAO) Educational Resources

Website: https://elearning.fao.org/ (accessed on 29 May 2026).

The FAO of the United Nations provides extensive educational resources focused on AMR within the context of food systems, livestock production, and agriculture. Through its eLearning platform, it offers self-paced courses, training materials, technical guides, and awareness-raising resources aimed at professionals, policymakers, farmers, and educators. These materials emphasize the reduction in antimicrobial use in animal production, the promotion of responsible practices, and the strengthening of surveillance systems in food and agriculture sectors.

A key feature of the FAO’s resources is their alignment with the OH approach, integrating human, animal, and environmental health perspectives. This makes them particularly valuable for designing interdisciplinary educational interventions and for supporting capacity building in low- and middle-income countries.

#### 4.6.3. World Organization for Animal Health (WOAH) OH Resources

Resources include international OH guidance, operational tools, workforce development materials, and multisectoral AMR resources from the human, animal, and environment interface.

#### 4.6.4. Tripartite OH Zoonoses Guide (WHO–FAO–WOAH)

Collaborative guidance document supporting countries in implementing OH approaches for zoonoses, food safety, and antimicrobial resistance management.

#### 4.6.5. The Fleming Fund Programme

This UK aid programme developed a comprehensive online curriculum on AMR in partnership with the Open University. It features a dedicated Veterinary Services learning pathway that covers everything from epidemiological surveillance of resistant bacteria on farms to correct clinical sampling techniques.

## 5. Pedagogical Approaches and Best Practices

### 5.1. Active Learning Approaches

The most effective AMR educational programmes employ active learning strategies that engage students as active participants rather than passive recipients of information. Active learning methodologies have demonstrated significant benefits in STEM disciplines compared to traditional approaches. A meta-analysis of 225 studies revealed that active learning methods consistently achieve better educational outcomes in STEM disciplines [58].

The methodologies employed in AMR educational programmes are as follows.

#### 5.1.1. Service-Learning (SL)

SL is considered a comprehensive pedagogical approach that combines academic learning with community service, promoting both personal development and civic responsibility [59]. It is an experiential methodology that unites learning and community service in a single project with an academic and civic foundation [60]. This method not only aims for students to acquire theoretical knowledge but also actively involves them in solving community problems, which contributes to their development as responsible and engaged citizens. Service-learning (SL) fosters a cycle of care and empowerment in young people, allowing them to practice democratic citizenship skills from primary to secondary school [61]. This approach not only benefits students in terms of personal and academic development but also strengthens their connection to the community, as evidenced by the fact that young people who participate in service-learning experiences tend to demonstrate greater community engagement in the future [62]. Furthermore, research suggests that opportunities to reflect on these experiences are crucial, as they allow students to link theoretical learning with real-world practice [63]. On the other hand, SL has been observed to be an effective bridge between theory and practice, especially in teacher training, as it provides educators not only with pedagogical skills but also with a deeper understanding of the social realities their future students face [64]. This approach reflects the importance of interdisciplinary training and experiential learning, which are crucial for preparing students for real-world challenges [65]. Implementing service-learning programmes across different disciplines has proven effective in improving student motivation and academic performance [66]. Ultimately, service-learning emerges as a decisive educational strategy for developing an active and engaged citizenry capable of addressing contemporary challenges.

MicroMundo is an SL educational programme that combines academic learning, citizen science, and community engagement. An evaluation of the MicroMundo programme involved pre- and post-intervention questionnaires and satisfaction surveys administered to 137 secondary school students and 14 teachers. Results showed statistically significant improvements in knowledge related to antibiotics, antimicrobial resistance, its health and environmental consequences, and measures to mitigate the problem across all educational levels. In addition, participants reported increased interest in science and research careers, suggesting that the project not only enhanced AMR literacy but also promoted scientific vocations. These findings support the effectiveness of SL as a pedagogical strategy for improving AMR-related knowledge while simultaneously engaging students in a meaningful scientific service to society [67].

#### 5.1.2. Experiential and Inquiry-Based Learning

Experiential learning is based on the principle that knowledge is constructed through direct experience and reflection. In AMR education, experiential and inquiry-based approaches frequently employ hands-on laboratory activities that allow learners to investigate microbiological phenomena firsthand. Programmes such as Tiny Earth engage students in authentic antibiotic discovery research, including soil sampling, bacterial isolation, screening for antimicrobial activity, and data sharing within a global research network. Recent evidence from the Tiny Earth programme showed significant increases in students’ scientific self-efficacy and scientific identity, with gains observed across diverse student populations and educational contexts [68].

Similarly, e-Bug incorporates practical classroom activities and laboratory experiments that help students visualize microbial transmission, hygiene practices, and the role of antibiotics. Evaluations of the programme have demonstrated positive effects on knowledge acquisition, retention, and educator capacity building across school, professional, and community settings. The original e-Bug school resource employed a curriculum-integrated, teacher-led educational intervention consisting of lesson plans, background materials, and interactive classroom activities on microbes, hygiene, infection prevention, and prudent antibiotic use. Using a quasi-experimental design with intervention and control groups, Lecky et al. reported significant improvements in knowledge among primary school students (9–11 years) across all educational domains, with no significant decline in scores six weeks after teaching, indicating sustained knowledge retention. Among secondary school students (12–15 years), knowledge gains were also observed, although results varied across participating countries (England, France, and the Czech Republic) [69].

#### 5.1.3. Game-Based Learning

Game-based learning (GBL) is based on engagement and motivation theories, highlighting the role of enjoyment and deep learning and increased motivation [70]. GBL employs complete, often stand-alone, games designed to achieve specific learning objectives. In science education, digital game-based learning is the most common and is linked to better content understanding, greater scientific achievement, and faster task completion [71]. Simulation games like Micro-Combat allow students to experience the challenges of decision-making in managing antibiotic use and resistance. These active approaches are particularly effective for developing systems thinking and understanding the complexity of OH challenges; however, published evidence evaluating Micro-Combat educational effectiveness remains limited.

#### 5.1.4. Gamification

Gamification integrates game elements into existing curriculum structures to increase engagement. Gamification elements, such as progress tracking, points, and achievement badges, foster intrinsic motivation and encourage continued engagement with educational content [72]. Resources from EducaCaixa and Ambientech demonstrate how interactive animations, interactive diagrams, and game mechanics can make learning about microscopic processes and epidemiological concepts more accessible and engaging, although evidence regarding its impact on AMR-related learning outcomes is currently lacking.

#### 5.1.5. Interdisciplinary and Systems Thinking-Based Learning (ST)

The OH approach to AMR inherently requires interdisciplinary thinking. Science educators have highlighted both the importance and the difficulty of developing ST to address complex health challenges such as AMR. Effective educational strategies are needed to support this development to integrate multiple perspectives when interpreting health-related systems.

In this sense, recent research examining modelling activities with 56 pre-service teachers found that such approaches can foster ST development in the context of AMR. Participants engaged in written explanations and group activities designed to promote the identification of system components and relationships, the proposal of actions, and the adoption of an “inside the system” perspective. After completing the sequence, a majority of students incorporated aspects of the OH vision in their explanations. However, the degree of ST achieved was not uniform across participants, highlighting the complexity of developing these competencies and the need for further research on their long-term consolidation. Notably, multimodal representations used during intermediate activities appeared particularly effective in supporting this type of reasoning. The study concludes that the use of diverse representational formats can facilitate ST development and improve understanding of complex health systems [73].

Nevertheless, these findings should be interpreted with caution. The study was conducted with a relatively specific sample of pre-service teachers, and the reported improvements do not necessarily guarantee the transfer of ST skills to other contexts or their long-term retention. Further research is needed to explore the sustainability, transferability, and generalizability of these learning outcomes.

This systems perspective contrasts with traditional, compartmentalized approaches to health education and is essential for developing the holistic understanding needed to address global challenges such as AMR. The integration of agricultural, environmental, clinical, social, economic, and political dimensions within OH education reflects this interdisciplinary framework.

Programmes can foster ST through activities such as multimodal representations, concept maps, causal loop diagrams, or role-playing that simulates the interactions among different stakeholders in the AMR system.

### 5.2. Real-World and Context-Based Learning

Effective AMR education connects abstract concepts to learners’ lived experiences and local contexts. Context-based science education has been shown to improve student engagement, motivation, and the meaningful understanding of scientific concepts by linking learning to real-world situations and socio-scientific issues [74,75]. In the context of AMR education, this approach is particularly relevant because antimicrobial resistance constitutes a complex socio-scientific issue that directly affects public health, food systems, and environmental sustainability [76].

Programmes that relate AMR to familiar infectious diseases, everyday antibiotic use, and real-world health challenges can increase the relevance of learning and promote meaningful engagement with the topic. Educational resources such as e-Bug contextualize concepts related to microbiology, infection prevention, hygiene, and prudent antibiotic use through situations that are familiar to learners. Evaluation studies have shown that participation in e-Bug activities improved students’ knowledge of microbes, hygiene, and antibiotics, with evidence of sustained knowledge retention following the intervention [69]. By linking scientific concepts to issues that directly affect individuals and communities, context-based approaches help learners understand the personal and societal consequences of AMR, including the risk of common infections becoming difficult to treat and the broader public health and economic impacts of antimicrobial resistance. Such approaches can support informed decision-making regarding antibiotic use and encourage greater awareness of antimicrobial stewardship.

### 5.3. Behaviour Change Focus

Beyond knowledge transmission, effective AMR education should aim to influence attitudes and behaviours. Educational interventions addressing antimicrobial resistance increasingly draw on behaviour change theories, particularly the Theory of Planned Behaviour [77] and Social Cognitive Theory [78], which emphasize the importance of attitudes, perceived norms, self-efficacy, and behavioural intentions in promoting responsible health practices.

Programmes should explicitly address common misconceptions, such as the belief that antibiotics are effective against viral infections, normalize appropriate health-seeking behaviours, and empower learners to take action through proper antibiotic use, infection prevention, and advocacy for policy changes. Research shows that public understanding of antimicrobial resistance remains limited and is frequently associated with misconceptions regarding antibiotic use [79].

## 6. Current Challenges and Gaps

### 6.1. Curriculum Integration

As discussed in Section 3.2, international OH policies strongly advocate the integration of AMR and OH concepts into formal education. However, translating these policy recommendations into routine educational practice remains challenging. Institutional barriers, including rigid curriculum structures, competing educational priorities, and limited flexibility to introduce interdisciplinary content, often hinder implementation. Organisational constraints, such as insufficient coordination between education and health authorities, together with financial limitations affecting teacher training, laboratory resources, and curriculum development, further reduce the sustainability and scalability of AMR educational initiatives.

### 6.2. Teacher Training and Resources

Teachers require both content knowledge about AMR and OH and pedagogical skills for teaching these topics effectively. Many educators report insufficient preparation to teach AMR, particularly the molecular biology, epidemiology, and interdisciplinary aspects. Professional development programmes specifically focused on AMR education remain limited [80].

Addressing this gap requires systematic professional development opportunities, including workshops, online courses, and continuing education programmes for in-service teachers [81]. Partnerships between educational institutions and research organisations like ISGlobal can provide teachers with access to current scientific knowledge and resources. Pre-service teacher education programmes should also incorporate AMR and OH content. The OH Joint Plan of Action also highlights workforce development and interdisciplinary education as key priorities for strengthening global responses to AMR and other interconnected health threats [82].

### 6.3. Laboratory Access and Resources

Hands-on microbiology activities are highly effective for teaching AMR but require laboratory facilities, equipment, and materials that many schools lack, particularly in resource-limited settings. This creates inequities in access to high-quality AMR education.

Solutions include developing low-cost alternatives to traditional laboratory activities, such as simplified culturing techniques using household materials; creating virtual laboratory simulations; establishing partnerships with local universities or hospitals to provide laboratory access; and prioritizing resource allocation to support science laboratory infrastructure. In this line, it is worth highlighting the MicroMundo project, in which the materials used in secondary education centres are provided by universities, making it possible to carry out laboratory practices that would otherwise be unfeasible within the budget constraints of a secondary school [34].

### 6.4. Evaluation and Evidence Base

While many AMR educational programmes exist, rigorous evaluation of their effectiveness remains limited. Few programmes have published peer-reviewed assessments of knowledge gains, attitude changes, or behavioural impacts. Without robust evaluation data, it is difficult to identify which approaches are most effective and to justify continued investment in educational interventions.

Some educational initiatives have undergone formal evaluation. For example, the e-Bug programme [69] has consistently demonstrated improvements in students’ knowledge of microbes, infection prevention, and prudent antibiotic use across several European countries, while the MicroMundo/Tiny Earth initiatives [67,68] have reported gains in microbiology knowledge, scientific competencies, and awareness of AMR among secondary school and university students. However, these evaluations frequently rely on pre-post study designs without control groups, involve relatively small or self-selected samples, and assess mainly short-term knowledge acquisition rather than long-term behavioural change. These methodological limitations make it difficult to compare interventions or determine their sustained impact.

In contrast, many international training programmes developed by organisations such as WHO, FAO, and WOAH have primarily reported indicators related to participation, course completion, and capacity building, while formal evaluations of their long-term educational impact remain scarce. A similar situation applies to molecular epidemiology training programmes, which have strengthened technical competencies in AMR surveillance but have rarely been assessed with standardized educational outcome measures. This highlights the need for more robust and comparable evaluation frameworks across different types of AMR educational initiatives.

The predominance of European initiatives identified in this review may partly reflect the greater availability of published and formally evaluated educational programmes rather than the absence of similar initiatives in other regions. Nevertheless, more studies evaluating OH-based AMR education in low- and middle-income countries are needed to strengthen the global evidence base and improve the international applicability of current educational recommendations.

Future research should therefore prioritize robust evaluation frameworks using standardized assessment tools, longitudinal follow-up, and, where feasible, controlled study designs. Such approaches would facilitate comparisons across educational settings and strengthen the evidence base for scaling effective AMR education programmes.

### 6.5. Limited Integration of Environmental Dimension

Despite growing recognition of environmental factors in AMR transmission, many educational programmes still provide limited coverage of this dimension. The role of environmental contamination, wastewater treatment, pharmaceutical residues, and ecological reservoirs of resistance genes often receive less attention than clinical and agricultural aspects. Strengthening the environmental component of OH AMR education represents an important gap to address in future programmes [73].

Several authors have warned that the environmental dimension continues to be the “neglected component” of the OH framework despite its major role in the dissemination of resistance genes and antimicrobial residues [82].

## 7. Recommendations for Strengthening AMR Education

Among the recommendations presented below, three priorities emerge as essential for the effective implementation of One Health-based AMR education: (i) the integration of AMR and OH into national curricula, (ii) sustained teacher training and professional development, and (iii) the establishment of robust evaluation frameworks to assess educational outcomes. These priorities provide the structural foundation upon which the remaining recommendations can be effectively implemented.

### 7.1. Policy and Curriculum

The highest priority should be the explicit integration of AMR and OH concepts into national education curricula, as curriculum inclusion provides the foundation for all subsequent educational actions. National education authorities should explicitly include AMR and OH topics in science and health education curriculum standards. This inclusion should span multiple grade levels with age-appropriate progression from basic concepts of infection and hygiene in primary education to a more sophisticated understanding of resistance mechanisms, epidemiology, and systems thinking in secondary and tertiary education.

Standardized assessments should include items related to AMR to signal the importance of this content and ensure accountability for teaching it. Curriculum developers should provide clear guidance on integrating AMR education within existing subject areas, including biology, health sciences, environmental science, and social studies. International organisations increasingly advocate the integration of sustainability and systems thinking competencies into formal education as essential components for addressing complex global challenges such as AMR [83]. The success of initiatives such as e-Bug demonstrates that curriculum-aligned educational resources can improve students’ knowledge of infection prevention and prudent antibiotic use when implemented systematically within schools [69].

### 7.2. Teacher Support and Professional Development

Once curricular integration is achieved, sustained teacher professional development should be considered the second key priority, as teachers are the main agents responsible for translating curriculum objectives into classroom practice.

Comprehensive professional development programmes should equip teachers with both content knowledge and pedagogical strategies for effective AMR education. These programmes should emphasize active learning approaches, OH perspectives, and strategies for addressing common student misconceptions.

Professional development should be ongoing rather than one-time events, with opportunities for continued learning and peer collaboration. Online platforms and professional learning communities can facilitate sustained support. Pre-service teacher education programmes should incorporate AMR and OH content to ensure new teachers enter the profession prepared to teach these topics.

Developing systems thinking competencies among teachers is particularly important because understanding AMR requires the ability to interpret dynamic and interconnected biological, environmental, social, and political systems [73,83].

Experiences from programmes such as MicroMundo have also shown that collaboration between researchers and teachers enhances both scientific accuracy and student engagement [67].

### 7.3. Resource Development and Dissemination

To support curriculum implementation, continued investment in developing high-quality, evidence-based educational resources is essential. Resources should be freely accessible, adaptable to local contexts, and available in multiple languages. Digital platforms like e-Bug and Ambientech exemplify accessible, comprehensive resources that can be widely disseminated.

Resource repositories should be well-curated and easily searchable, allowing educators to quickly find materials suitable for their specific needs. Quality assurance mechanisms should ensure that resources reflect current scientific understanding and adhere to pedagogical best practices.

### 7.4. Cross-Sectoral Collaboration

Effective AMR education requires collaboration between education, health, agriculture, and environment sectors. Partnerships should be established at national, regional, and local levels to coordinate messaging, sharing resources, and leverage diverse expertise. Initiatives such as e-Bug, Tiny Earth, MicroMundo, and collaborations between research institutions such as ISGlobal and educational foundations such as EduCaixa demonstrate the added value of cross-sectoral partnerships in translating OH principles into educational practice across different educational settings.

International organisations such as WHO, FAO, WOAH, and UNEP have established a strong policy framework for One Health-based AMR education through guidance documents, technical resources, and training programmes. However, greater efforts are still needed to fully integrate environmental health, food safety, and food system perspectives into educational programmes, as these dimensions often receive less attention than human and animal health. Future strategies should also strengthen interdisciplinary training by promoting collaboration among health, veterinary, agricultural, environmental, and educational professionals, while encouraging innovative educational approaches.

Professional organisations in medicine, veterinary science, agriculture, and education should prioritize AMR education in their activities, providing leadership and credibility to educational initiatives. Strengthening collaboration across these sectors will facilitate the exchange of expertise, the harmonization of educational resources, and the implementation of evidence-based educational practices, thereby supporting the translation of international One Health strategies into effective educational policies and programmes at national and local levels.

### 7.5. Research and Evaluation

Establishing rigorous evaluation frameworks should be considered a strategic priority for strengthening AMR education, as robust evidence is essential to identify effective educational approaches, inform educational policy, and justify sustained investment in educational programmes. A robust research agenda should investigate effective pedagogical approaches for teaching AMR and OH concepts, identify common learning challenges and misconceptions, and evaluate the impact of educational interventions on knowledge, attitudes, and behaviours. Research findings should inform the continuous improvement of educational programmes.

Evidence from existing initiatives illustrates the value of systematic evaluation. For example, the evaluation of the MicroMundo project demonstrated improvements in participants’ knowledge of AMR and helped identify the educational stages at which the intervention was most effective [67]. Similar evaluations of programmes such as e-Bug have also reported significant gains in students’ understanding of microorganisms, infection prevention, and responsible antibiotic use, highlighting the benefits of evidence-based educational interventions [69].

Mixed methods combining quantitative measures with qualitative insights can provide a comprehensive understanding of programme effectiveness. Evaluation frameworks should extend beyond immediate post-intervention assessments to examine longer-term outcomes, behavioural changes, and community-level impacts. Where feasible, standardized assessment tools and controlled or longitudinal study designs should be adopted to facilitate comparisons across educational settings and strengthen the evidence base for future AMR education programmes.

### 7.6. Equity and Access

Educational equity requires ensuring that high-quality AMR education reaches all learners regardless of geographic location, socioeconomic status, or educational setting. This includes developing low-cost alternatives to resource-intensive activities, providing materials in multiple languages, and addressing digital divides that might limit access to online resources.

Special attention should be given to resource-limited settings where AMR burdens are often highest but educational resources are most scarce. International development organisations and philanthropic entities can support AMR education initiatives in low- and middle-income countries.

Ensuring equitable access should be considered a cross-cutting principle underpinning all previous recommendations, particularly in low-resource settings where the burden of AMR is greatest.

## 8. Future Directions

Future educational programmes should strengthen the integration of environmental sciences, promote policy-oriented education, and leverage digital technologies for a broader reach. The development of standardized competencies and evaluation indicators for OH AMR education could enhance comparability and impact across different programmes and contexts. Future research should prioritize the development and validation of competency frameworks that define the knowledge, skills, and attitudes expected at different educational levels, together with standardized assessment instruments that allow comparisons across institutions and countries. In addition, comparative studies evaluating the effectiveness of different pedagogical approaches would provide valuable evidence to guide curriculum design and educational policy.

Embedding OH AMR education within national action plans and professional accreditation systems may contribute to sustainability. When AMR education becomes a requirement for professional licensure or certification, incentives align to ensure comprehensive coverage. The integration of emerging technologies, including mobile learning platforms, virtual reality for immersive laboratory experiences, and artificial intelligence for personalized learning pathways, holds promise for significantly expanding the reach and effectiveness of AMR education.

Finally, fostering international collaboration and knowledge exchange will be essential. Platforms for sharing educational resources, pedagogical innovations, and evaluation findings across countries and regions can accelerate progress and avoid duplication of efforts. The establishment of a global repository of OH AMR educational resources, coupled with communities of practice for educators and programme developers, could significantly advance the field. Such collaborative networks could also promote the development of common evaluation frameworks, multicentre educational research, and the exchange of best practices, thereby generating more robust evidence to inform both educational policy and future AMR strategies.

Overall, this review provides the first comprehensive synthesis of educational initiatives addressing AMR from an explicit OH perspective across formal, non-formal, and professional education. The findings identify key priorities for future research and educational programme development, including the establishment of standardized competency frameworks, the implementation of robust evaluation methodologies, the stronger integration of environmental and food system perspectives, and the generation of evidence from low- and middle-income countries. Collectively, these priorities provide a roadmap to support educators, researchers, policymakers, and international organisations in the design, implementation, evaluation, and continuous improvement of effective OH-based AMR education programmes.

## Figures and Tables

**Table 1:** Comparative characteristics of educational programs and resources addressing AMR through a OH approach.

Program	Target Audience	Intervention Type	OH Score *	Pedagogical Methods	Educational Approach	Impact Assessment Data (Source)
**e-Bug**	Primary–Secondary	Curriculum-based program	3	Laboratory activities, worksheets, interactive lessons	Experiential learning	Studies report significant improvements in knowledge and attitudes (peer-reviewed publications)
**ISGlobal Programs**	Secondary–Community	Serious games and outreach activities	3	Gamification, multimedia resources	Serious games	Limited formal evaluation reported; recognition through awards and outreach reach
**Ambientech**	Secondary–Vocational	Interactive online platform	4	Gamification, problem-solving, case studies	Gamified learning	No specific impact data reported
**Tiny Earth**	Secondary–University	Citizen science research program	3	Authentic research and laboratory work	Citizen science	Educational and research outcomes reported
**MicroMundo**	Secondary–University	Citizen science and service learning	4	Mentoring and collaborative research	Citizen science + service learning	Formal impact evaluation reported (peer-reviewed publications)
**PRAN School Initiatives**	Primary–Secondary	Curriculum integration and teacher training	3	Workshops and classroom resources	Public health education	No specific evaluation data reported
**DivulSuperbac**	Secondary–University	Service-learning outreach program	2	Infographics and peer education	Service learning	Post survey evaluation questionnaires reported (peer-reviewed publications)
**OAZIS Health**	Community	Online course	3	E-learning and behaviour-change education	Community capacity building	No evaluation data reported
**University Activities (PARE, AntibioGame®, etc.)**	University	Research, laboratory and game-based learning	2–4	Simulations, laboratory research, role-play	Research-based learning	Educational effectiveness reported in literature
**ESCMID Course**	Professionals	Online professional training	2	Self-directed online learning	Continuing professional education	No impact data reported
**AMR EDUCare**	Professionals	Modular online training	2	Competency-based learning	European training network	No impact data reported
**PRAN–UCM Course**	Professionals	Continuing education course	4	Lectures and interdisciplinary discussion	OH professional training	No formal evaluation reported
**Coursera AMR**	Students–Professionals	MOOC	3	Videos, quizzes, self-paced learning	Global online learning	Reviews, ratings, number of subscribers, satisfaction percentage (platform analytics)
**FAO eLearning Academy**	Professionals–Policymakers	Online capacity-building courses	4	Case studies and policy-oriented learning	International OH training	Course evaluation forms, badges/certification, number of participants, institutional reach (official training webpages/evaluation forms)
**TGHN AMR Portal**	Professionals	Online training portal	3	Modular multilingual learning	Global collaborative learning	Public impact page of the portal (institutional impact webpage)
**OHWA**	Professionals–Educators	Online training platform	4	Applied learning and assessments	Workforce development	No impact data reported
**ISCAID**	Veterinary professionals	Webinars, workshops and case-based training	2	Clinical cases and expert-led sessions	Veterinary antimicrobial stewardship	No formal educational impact data reported
**WSAVA Academy**	Veterinary professionals	Online and hybrid training modules	2	Guideline-based learning and clinical cases	Veterinary continuing education	No formal educational impact data reported
**WHO-FAO-WOAH Zoonotic Disease Outbreak Response Course**	Professionals-Policymakers	Joint online training course	4	Scenario-based and coordinated response training	OH outbreak preparedness	Course completion/certification data available; limited impact assessment reported
**ESAVS**	Veterinary professionals	Postgraduate veterinary education	2	Specialty-based modules and clinical training	Veterinary professional development	No specific AMR education impact data reported
**WHO Educational Resources**	Professionals-Policymakers-Educators	Training modules, webinars and policy resources	3	Self-directed learning and technical guidance	Global public health education	Institutional reach and training completion data; limited formal impact assessment
**FAO Educational Resources**	Professionals-Policymakers-Farmers-Educators	E-learning, technical guides and awareness resources	4	Case studies, policy guidance and applied learning	Food systems and agricultural AMR education	Course evaluation forms, certification/badges and participation data; limited long-term impact evidence
**WOAH OH Resources**	Veterinary and public health professionals	Guidance documents, tools and training materials	3	Operational tools and technical guidance	Animal health and OH capacity building	Institutional capacity-building indicators; limited formal educational impact data
**Tripartite OH Zoonoses Guide**	Policymakers-Professionals	Guidance document and operational tools	4	Multisectoral coordination and applied guidance	OH policy and implementation training	No formal educational impact data reported
**Fleming Fund Program**	Laboratory and surveillance professionals	Online curriculum and technical training	3	Learning pathways, surveillance methods and sampling practice	AMR surveillance and molecular epidemiology training	Capacity-building and training outputs reported; limited long-term outcome evaluation

* OH Score: OH Integration Score: 1 = primarily focused on human health; 2 = limited integration of additional OH dimensions; 3 = explicit integration of multiple OH dimensions; 4 = OH serves as the central conceptual framework, integrating human, animal, plant, environmental, and food system perspectives where appropriate.

## Data Availability

No new data were created or analyzed in this study. Data sharing is not applicable to this article.

## Abbreviations

The following abbreviations are used in this manuscript:

AMR	Antimicrobial resistance
OH	One Health
ST	Systems thinking

## References

[1] World Health Organization (WHO) (2014). Global Action Plan on Antimicrobial Resistance.

[2] O’Neill J. (2014). Antimicrobial resistance: Tackling a crisis for the health and wealth of nations. The Review on Antimicrobial Resistance.

[3] Plan Nacional Frente a La Resistencia a Los Antibióticos (PRAN). https://resistenciaantibioticos.es.

[4] World Health Organization (2023). Antimicrobial Resistance.

[5] Laxminarayan R., Duse A., Wattal C., Zaidi A.K.M., Wertheim H.F.L., Sumpradit N., Vlieghe E., Hara G.L., Gould I.M., Goossens H. (2013). Antibiotic resistance-the need for global solutions. Lancet Infect. Dis..

[6] McEwen S.A., Collignon P.J., Schwarz S., Cavaco L.M., Shen J. (2018). Antimicrobial resistance: A One Health perspective. Antimicrobial Resistance in Bacteria from Livestock and Companion Animals.

[7] Hernando-Amado S., Coque T.M., Baquero F., Martínez J.L. (2019). Defining and combating antibiotic resistance from One Health and global health perspectives. Nat. Microbiol..

[8] Velazquez-Meza M.E., Galarde-López M., Carrillo-Quiróz B., Alpuche-Aranda C.M. (2022). Antimicrobial resistance: One Health approach. Vet. World.

[9] Robinson T.P., Bu D.P., Carrique-Mas J., Fèvre E.M., Gilbert M., Grace D., Hay S.I., Jiwakanon J., Kakkar M., Kariuki S. (2016). Antibiotic resistance is the quintessential One Health issue. Trans. R. Soc. Trop. Med. Hyg..

[10] (2022). One Health High-Level Expert Panel (OHHLEP). One Health: A new definition for a sustainable and healthy future. PLoS Pathog..

[11] Collignon P., Beggs J.J., Walsh T.R., Gandra S., Laxminarayan R. (2018). Anthropological and socioeconomic factors contributing to global antimicrobial resistance: A univariate and multivariable analysis. Lancet Planet. Health.

[12] Bengtsson-Palme J., Kristiansson E., Larsson D.J. (2018). Environmental factors influencing the development and spread of antibiotic resistance. FEMS Microbiol. Rev..

[13] Jesumirhewe C., Odufuye T.O., Ariri J.U., Adebiyi A.T., Sanusi A.T., Stöger A., Daza-Prieto B., Allerberger F., Cabal-Rosel A., Ruppitsch W. (2024). Genetic characterization of antibiotic-resistant *Staphylococcus* spp. and *Mammaliicoccus sciuri* from healthy humans and poultry in Nigeria. Antibiotics.

[14] Rybak B., Jarzembowski T., Daca A., Krawczyk B., Piechowicz L. (2025). Genetic determinants and biofilm properties useful in estimation of UTI pathogenicity of the *Escherichia coli* strains isolated from free-living birds. Antibiotics.

[15] Fusaro C., Miranda-Madera V., Serrano-Silva N., Bernal J.E., Ríos-Montes K., González-Jiménez F.E., Ojeda-Juárez D., Sarria-Guzmán Y. (2024). Antibiotic-resistant bacteria isolated from street foods: A systematic review. Antibiotics.

[16] Drane K., Sheehan M., Whelan A., Ariel E., Kinobe R. (2024). The role of wastewater treatment plants in dissemination of antibiotic resistance: Source, measurement, removal and risk assessment. Antibiotics.

[17] Moza A.-C., Bucur I.-M., Imre K., Popa S.A., Grigoreanu A.A., Plotuna A.-M., Ivan A.A., Mederle N.G., Tîrziu A.-T., Tîrziu E. (2026). Phenotypic antimicrobial resistance of some bacterial strains isolated from red foxes (*Vulpes vulpes*) in Western Romania. Antibiotics.

[18] Díez de los Ríos J., Párraga-Niño N., Navarro M., Serra-Pladevall J., Vilamala A., Arqué E., Baldà M., Blanco T.N., Pedro-Botet L., Mascaró O. (2025). Environmental dispersion of multiresistant Enterobacteriaceae in aquatic ecosystems in an area of Spain with a high density of pig farming. Antibiotics.

[19] Touati A., Ibrahim N.A., Mairi A., Kirat H., Basher N.S., Idres T. (2025). One Health at risk: Plasmid-mediated spread of mcr-1 across clinical, agricultural, and environmental ecosystems. Antibiotics.

[20] Veloo Y., Syed Abu Thahir S., Zakaria Z., Abdul Rahman S., Mansor R., Rajendiran S. (2025). A scoping review unveiling antimicrobial resistance patterns in the environment of dairy farms across Asia. Antibiotics.

[21] Nowbuth A.A., Asombang A.W., Alaboud K., Souque C., Dahu B.M., Pather K., Mwanza M.M., Lotfi S., Parmar V.S. (2023). Gamification as an educational tool to address antimicrobial resistance: A systematic review. JAC-Antimicrob. Resist..

[22] López-Pintor E., Gómez-Ramos A., Sanz-Valero J. (2023). Antibiotic infographics available on the internet: Documentary quality, purpose, and appropriateness as educational tools on antimicrobial resistance. Antibiotics.

[23] Fuller W., Kapona O., Aboderin A.O., Adeyemo A.T., Olatunbosun O.I., Gahimbare L., Ahmed Y.A. (2023). Education and awareness on antimicrobial resistance in the WHO African region: A systematic review. Antibiotics.

[24] Allerton F., Russell J. (2023). Antimicrobial stewardship in veterinary medicine: A review of online resources. JAC-Antimicrob. Resist..

[25] Calvo-Villamañán A., San Millán Á., Carrilero L. (2023). Tackling AMR from a multidisciplinary perspective: A primer from education and psychology. Int. Microbiol..

[26] FAO, UNEP, WHO, WOAH (2022). One Health Joint Plan of Action (2022–2026).

[27] Iskandar K., Molinier L., Hallit S., Sartelli M., Hardcastle T.C., Haque M., Lugova H., Dhingra S., Sharma P., Islam S. (2021). Surveillance of antimicrobial resistance in low- and middle-income countries: A scattered picture. Antimicrob. Resist. Infect. Control.

[28] Rabinowitz P.M., Natterson-Horowitz B.J., Kahn L.H., Kock R., Pappaioanou M. (2017). Incorporating One Health into medical education. BMC Med. Educ..

[29] Kahn L.H. (2017). Antimicrobial resistance: A One Health perspective. Trans. R. Soc. Trop. Med. Hyg..

[30] Kilstadius M., Gericke N. (2017). Defining contagion literacy: A Delphi study. Int. J. Sci. Educ..

[31] Peel A., Zangori L., Friedrichsen P., Hayes E., Sadler T. (2019). Students’ model-based explanations about natural selection and antibiotic resistance through socio-scientific issues-based learning. Int. J. Sci. Educ..

[32] Bohlin G., Göransson A., Höst G.E., Tibell L.A.E. (2018). Insights from introducing natural selection to novices using animations of antibiotic resistance. J. Biol. Educ..

[33] Wanford J., Aidley J., Bayliss C., Ketley J., Goodwin M. (2018). Simulating phase variation: A practical approach to teaching mutation and diversity. J. Biol. Educ..

[34] Robredo B., Fernández-Fernández R., Torres C. (2023). Antimicrobial resistance as a nexus between teaching and research. J. Biol. Educ..

[35] Marvasi M., Casillas L., Vassallo A., Purchase D. (2021). Educational activities for students and citizens supporting the One-Health approach on Antimicrobial Resistance. Antibiotics.

[36] Alzard S., Exintaris B., Sarkar M., Grieve A., Chuang S., Coetzee R., Lim A. (2024). A global investigation into antimicrobial knowledge in medicine, pharmacy, nursing, dentistry, and veterinary undergraduate students: A scoping review to inform future planetary health multidisciplinary education. BMC Med. Educ..

[37] Drymiotou I., Quattrocchi A., Volkan E., Ioannides A., Alon-Ellenbogen D., Mavrides D., Ierodiakonou D., Gentekaki E., Salameh P., Karayiannis P. (2025). Open Schooling to raise student awareness and engagement: The case of tackling Antimicrobial Resistance. J. Biol. Educ..

[38] Ley Orgánica 3/2020, de 29 de Diciembre, por La Que se Modifica La Ley Orgánica 2/2006, de 3 de Mayo, de Educación. Boletín Oficial del Estado, 340, de 30 de Diciembre de 2020. https://www.boe.es/eli/es/lo/2020/12/29/3.

[39] Robredo B., Torres C. (2021). Are Secondary School Students Aware of the Pathogenicity of Microorganisms and the Problem of Antibiotic Resistance?. Rev. Eureka Sobre Enseñanza y Divulg. Las Cienc..

[40] Nadar S., Brown J.C., Coe L.S., Koukoulidis N.M., Czyż E.M., Czyż D.M. (2025). Antimicrobial resistance and One Health in the high school biology curriculum. J. Microbiol. Biol. Educ..

[41] Gil-Serna J., Antunes P., Campoy S., Cid Á., Cobo-Molinos A., Durão P. (2025). & all members of MicroMundo Teams in Spain and Portugal. Citizen science to raise antimicrobial resistance awareness in the community: The MicroMundo Project in Spain and Portugal. Microb. Biotechnol..

[42] Maicas S., Fouz B. (2024). DIVULSUPERBAC: An outreach project to raise awareness of antimicrobial resistance. FEMS Microbiol. Lett..

[43] Kupis K., Fouz B., Maicas S., Sobieraj I. (2025). The effectiveness of the service-learning method: A case study of the international ‘Superbugs’ Project. Health Educ. J..

[44] Ahmed R., Bashir A., Brown J.E.P., Cox J.A.G., Hilton A.C., Hilton C.E., Lambert P.A., Theodosiou E., Tritter J.Q., Watkin S.J. (2020). The drugs don’t work: Evaluation of educational theatre to gauge and influence public opinion on antimicrobial resistance. J. Hosp. Infect..

[45] Swe M.M.M., Hlaing P.H., Phyo A.P., Aung H.H., Smithuis F., Ashley E.A., Cheah P.Y. (2020). Evaluation of the forum theatre approach for public engagement around antibiotic use in Myanmar. PLoS ONE.

[46] Zhang Y., Kabba J., Chang J., Ji W., Zhu S., Yu J., Xu S., Fang Y. (2018). A School-Based Educational Intervention for School-Aged Children and Caregivers about Rational Use of Antibiotics in Urban Areas of Shaanxi Province: A Study Protocol for a Randomized Controlled Research. Int. J. Environ. Res. Public Health.

[47] Young V.L., Berry M., Verlander N.Q., Ridgway A., McNulty C.A. (2019). Using debate to educate young people in schools about antibiotic use and resistance: A before and after evaluation using a questionnaire survey. J. Infect. Prev..

[48] McNulty C.A.M., Brown C.L., Syeda R.B., Bennett C.V., Schofield B., Allison D.G., Francis N. (2020). Teacher and student views on the feasibility of peer to peer education as a model to educate 16–18 Year Olds on Prudent Antibiotic Use—A Qualitative Study. Antibiotics.

[49] Hall J., Jones L., Robertson G., Hiley R., Nathwani D., Perry M.R. (2020). ‘The Mould that Changed the World’: Quantitative and qualitative evaluation of children’s knowledge and motivation for behavioral change following participation in an antimicrobial resistance musical. PLoS ONE.

[50] Kvint K., Palm M., Farewell A. (2020). Teaching about antibiotic resistance to a broad audience: A multidisciplinary approach. FEMS Microbiol. Lett..

[51] Marvasi M., Choudhury M., Vala N.B., Teplitski M. (2017). Fitness of antibiotic-resistant bacteria in the environment: A laboratory activity. J. Microbiol. Biol. Educ..

[52] Popovich J., Stephens M., Celaya H., Suwarno S., Barclay S., Yee E., Dean D.A., Farris M., Haydel S.E. (2018). Building and Breaking the CellWall in Four Acts: A Kinesthetic and Tactile Role-Playing Exercise for Teaching Beta-Lactam Antibiotic Mechanism of Action and Resistance. J. Microbiol. Biol. Educ..

[53] Govindan B. (2018). Bacterial Survivor: An Interactive Game that Combats Misconceptions about Antibiotic Resistance. J. Microbiol. Biol. Educ..

[54] Fuhrmeister E.R., Larson J.R., Kleinschmit A.J., Kirby J.E., Pickering A.J., Bascom-Slack C.A. (2021). Combating Antimicrobial Resistance Through Student-Driven Research and Environmental Surveillance. Front. Microbiol..

[55] Tsopra R., Courtine M., Sedki K., Eap D., Cabal M., Cohen S., Bouchaud O., Mechaï F., Lamy J.-B. (2020). AntibioGame^®^: A serious game for teaching medical students about antibiotic use. Int. J. Med. Inform..

[56] Collignon P. (2013). The importance of a One Health approach to preventing the development and spread of antibiotic resistance. Curr. Top. Microbiol. Immunol..

[57] Harbarth S., Theuretzbacher U., Hackett J., Adriaenssens N., Anderson J., Antonisseet A., Årdal C., Baillon-Plot N., Baraldi E., Bettiol E. (2015). Antibiotic research and development: Business as usual?. J. Antimicrob. Chemother..

[58] Wieman C.E. (2014). Large-scale comparison of science teaching methods sends clear message. Proc. Natl. Acad. Sci. USA.

[59] Sigmon R.L. (1979). Service-learning: Three principles. Synergist.

[60] Sotelino A., Santos Rego M.A., Lorenzo M. (2016). Aprender y servir en la Universidad: Una vía cívica al desarrollo educativo. Teoría Educ..

[61] Terry A.W., Bohnenberger J.E. (2003). Service learning: Fostering a cycle of caring in our gifted youth. J. Second. Gift. Educ..

[62] Eyler J. (2002). Reflection: Linking service and learning—Linking students and communities. J. Soc. Issues.

[63] Waldstein F.A., Reiher T.C. (2001). Service-learning and students’ personal and civic development. J. Exp. Educ..

[64] Resch K., Schrittesser I. (2021). Using the service-learning approach to bridge the gap between theory and practice in teacher education. Int. J. Incl. Educ..

[65] Palpanadan S.T., Ahmad I., Isa K., Ravana V.K. (2020). Pedagogical implications of integrating service learning in engineering education. Int. J. Recent Technol. Eng..

[66] Martín-Sánchez M., González- Gómez D., Jeong J.S. (2022). Service Learning as an Education for Sustainable Development (ESD) teaching strategy: Design, implementation, and evaluation in a STEM university course. Sustainability.

[67] Robredo B., Fernández-Fernández R., Torres C., Ladrera R. (2023). MicroMundo: An experimental educational project fostering student engagement and knowledge on antibiotics and antimicrobial resistance in secondary education. FEMS Microbiol. Lett..

[68] Miller S., Hernandez P.R., Du W., Cervantes Aldana C., Lee H., Maldonado N., Sandoval P., Vong J., Young G., Handelsman J. (2025). Tiny Earth CURE Demonstrates Equitable Benefits for U.S. College Science Students. CBE—Life Sci. Educ..

[69] Lecky D.M., McNulty C.A.M., Adriaenssens N., Herotová T.K., Holt J., Kostkova P., Merakou K., Koncan R., Olczak-Pienkowska A., Avô A.B. (2010). Evaluation of e-Bug, an educational pack, teaching about prudent antibiotic use and hygiene, in the Czech Republic, France and England. J. Antimicrob. Chemother..

[70] Crocco F., Offenholley K., Hernandez C. (2016). A proof-of-concept study of game-based learning in higher education. Simul. Gaming.

[71] López-Fernández D., Gordillo A., Ortega F., Yagüe A., Tovar E. (2021). LEGO^®^ Serious Play in Software Engineering Education. IEEE Access.

[72] Lei H., Chiu M.M., Wang D., Wang C., Xie T. (2022). Effects of game-based learning on students’ achievement in science: A meta-analysis. J. Educ. Comput. Res..

[73] Uskola A., Gardeazabal H. (2026). Multimodal representations for developing preservice teachers’ systems thinking for addressing complex health issues. J. Balt. Sci. Educ..

[74] Bennett J., Lubben F., Hogarth S. (2007). Bringing science to life: A synthesis of the research evidence on the effects of context-based and STS approaches to science teaching. Sci. Educ..

[75] Gilbert J.K. (2006). On the nature of “context” in chemical education. Int. J. Sci. Educ..

[76] Sadler T.D. (2011). Socio-Scientific Issues in the Classroom: Teaching, Learning and Research.

[77] Ajzen I. (1991). The theory of planned behavior. Organ. Behav. Hum. Decis. Process..

[78] Bandura A. (2004). Health promotion by social cognitive means. Health Educ. Behav..

[79] McCullough A.R., Parekh S., Rathbone J., Del Mar C.B., Hoffmann T.C. (2016). A systematic review of the public’s knowledge and beliefs about antibiotic resistance. J. Antimicrob. Chemother..

[80] Dyar O.J., Huttner B., Schouten J., Pulcini C. (2017). What is antimicrobial stewardship?. Clin. Microbiol. Infect..

[81] Castro-Sánchez E., Moore L.S.P., Husson F., Holmes A.H. (2016). What are the factors driving antimicrobial resistance? Perspectives from a public event in London, England. BMC Infect. Dis..

[82] Essack S.Y. (2018). Environment: The neglected component of the One Health triad. Lancet Planet. Health.

[83] Arnold J.C., Wade J.P. (2015). A definition of systems thinking: A systems approach. Procedia Comput. Sci..

